# Fine Needle Aspiration Cytology in Diagnosis of Pure Neuritic Leprosy

**DOI:** 10.4061/2011/158712

**Published:** 2011-05-26

**Authors:** Bipin Kumar, Anju Pradhan

**Affiliations:** ^1^Department of Pathology, B. P. Koirala Institute of Health Sciences, Dharan, Nepal; ^2^Department of Pathology, Indira Gandhi Medical College & Research Institute, Puducherry 605009, India

## Abstract

Leprosy is a chronic infection affecting mainly the skin and peripheral nerve. Pure neuritic form of this disease manifests by involvement of the nerve in the absence of skin lesions. Therefore, it can sometimes create a diagnostic problem. It often requires a nerve biopsy for diagnosis, which is an invasive procedure and may lead to neural deficit. Fine needle aspiration cytology (FNAC) of an affected nerve can be a valuable and less invasive procedure for the diagnosis of such cases. We report five suspected cases of pure neuritic Hansen's disease involving the common and superficial peroneal, ulnar, and median nerve, who underwent FNAC. Smears revealed nerve fibers infiltrated by chronic inflammatory cells in all cases, presence of epithelioid cells granulomas, and Langhans giant cells in three cases, and acid fast bacilli in two cases. In conclusion, FNAC is a safe, less invasive, and time saving procedure for the diagnosis of pure neuritic leprosy.

## 1. Introduction

About 4–8% of all leprosy is clinically limited to the peripheral nerve [1]. This form of leprosy is termed pure neuritic leprosy [1]. The other names given are: neural, pure neural, primary neural, primary neuritic, purely neural, purely neuritic, or polyneuritic leprosy [1]. The clinical features of leprotic nerve involvement include nerve enlargement, tenderness, pain, and sensory motor impairment [1]. These are not specific and may not always be present [1]. The most commonly affected nerves include the posterior tibial, peroneal, ulnar, and median nerves [1]. Diagnosing leprosy in the absence of typical dermatological features is challenging and requires histological confirmation [1]. This is often achieved using nerve biopsy [1]. Limitations of this technique are sampling error, low sensitivity, and permanent nerve deficit [1]. A technique that is simpler than nerve biopsy is needed to evaluate the nerve involvement, especially in pure neuritic leprosy (PNL) [1]. Only a few studies have evaluated the role of fine needle aspiration cytology (FNAC) of the nerve in the diagnosis of PNL [2–7]. Here we report five cases of PNL diagnosed by FNAC.

## 2. Material and Methods

### 2.1. Cases

Five cases with varied complaining features and thickened nerves without any cutaneous lesion were subjected for FNAC from the department of dermatology of our hospital between the periods of October 2004 to December 2008.

### 2.2. Methods

The cases were examined for most prominent site of thickened nerve. The area was cleaned with an alcohol swab. The prominent part of nerve was fixed by index finger and thumb of left hand, and the 22 G needle fitted in 10 mL disposable plastic syringe was inserted along the length of the nerve. The suction was applied and aspiration was performed using a single-puncture, multidirectional technique. The direction of the needle was always kept parallel to the length of the nerve so as to cause minimal damage to the nerve. The material aspirated was smeared on glass slides. Minimum three smears were made for each case. The wet smear was fixed in 95% ethanol and stained by Papanicolaou stain after 30 minutes of fixation. One of the dried smear was stained by May-Grünwald-Giemsa (MGG) stain, and the other dried smear was stained by Fite's stain to demonstrate acid fast bacilli (AFB). All these smears were studied for cytological details.

### 2.3. Cytological Examination

Both Papanicolaou and MGG stained smears were examined for cellularity, presence of nerve fiber, Schwann cells and nerve fiber infiltration by inflammatory cells, lymphocytes, macrophages, epithelioid cells, granuloma, giant cells, and caseous necrosis. The cellularity was quantified into moderate (+) and good (++). Nerve fragments, schwann cells, and inflammatory cells are quantified according to the presence of their number, and it was denoted as, present (+), moderate in number (++) and numerous (+++). Smear stained by Fite's stain was examined for the presence or absence of AFB. If the AFB was seen, it was quantified according to the presence of their number per high-power field. It was denoted as present (+), if occasional bacilli was seen after searching it in many high-power fields, and many (++) if many bacilli per high-power field were seen. Negative finding was denoted as absent (−).

## 3. Result

**Figure 1:**
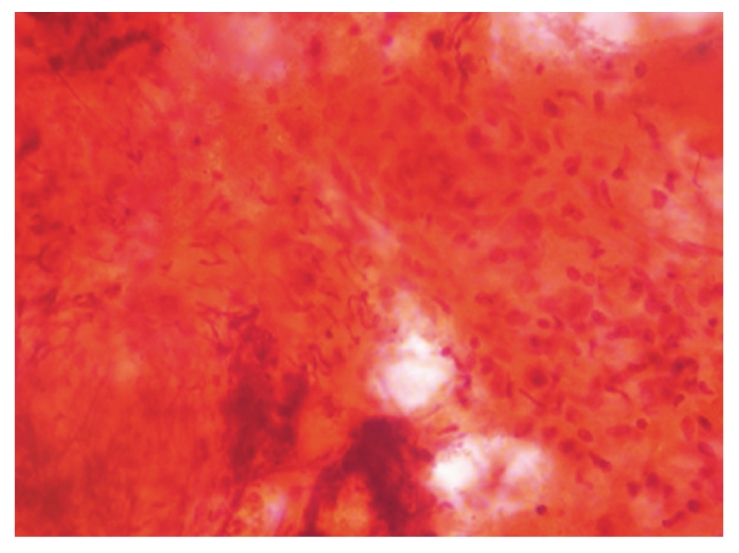
Nerve fragment showing infiltration by chronc inflammatory cells and granuloma (PAP, ×10).

**Figure 2:**
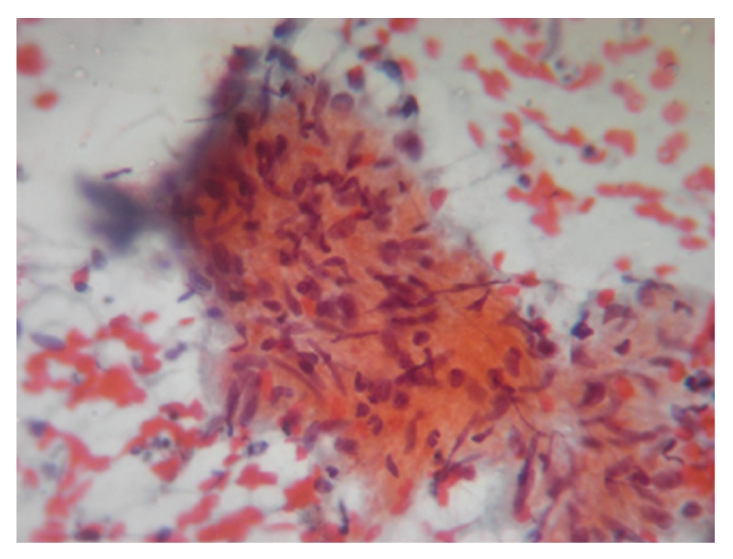
Spindle shaped Schwann cells infiltrated by chronc inflammatory cells comprising of macrophages, epithelioid cells and lymphocytes (PAP, ×40).

**Figure 3:**
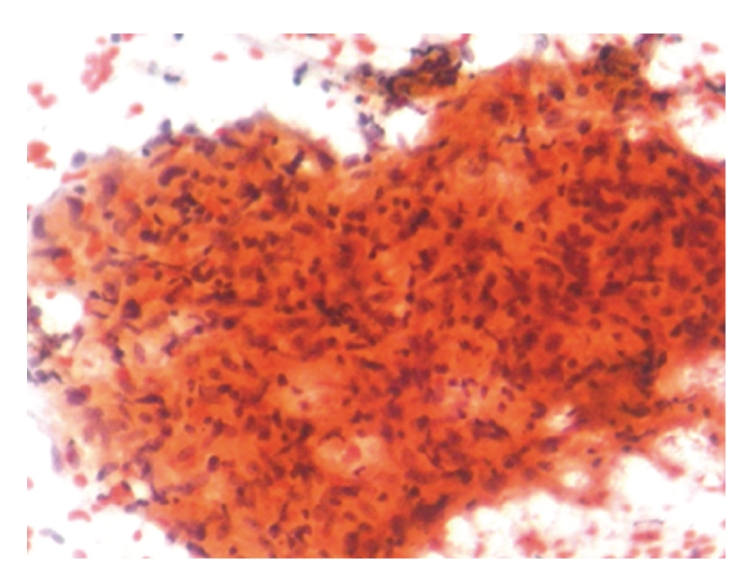
Smear showing epithelioid cells Granuloma (PAP, ×40).

**Figure 4:**
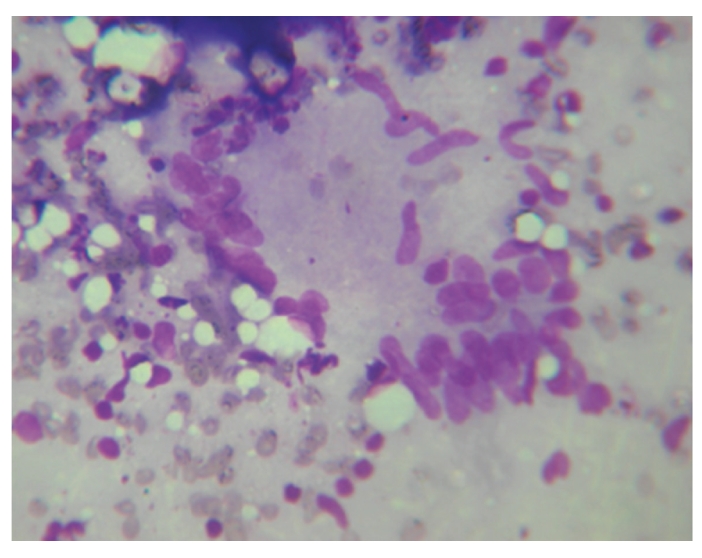
Smear showing Langhans giant cell (MGG, ×40).

**Figure 5:**
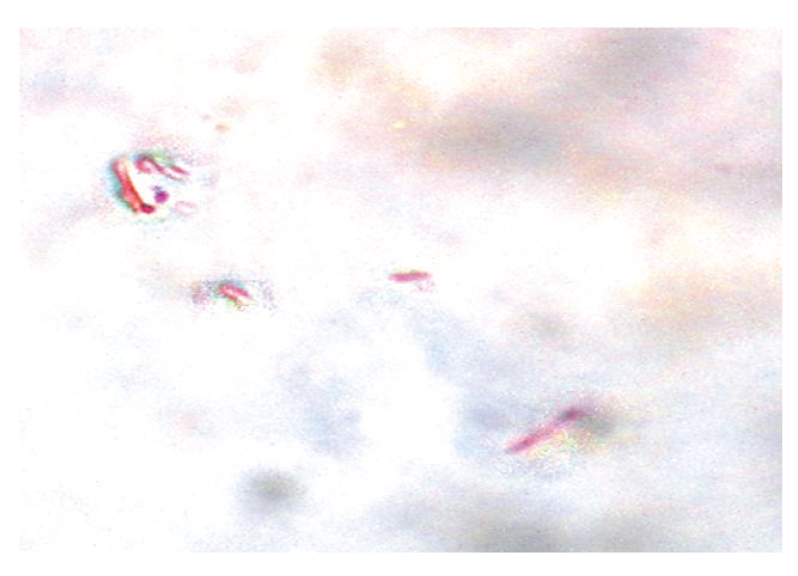
Smear showing AFB (Fite's Stain, Oil immersion).

All the cases had mononeuropathy. All the clinicocytological details of these cases have been compiled in Table 1. Out of 5 cases, 4 were male and one was female. The age range was 24–42 years. All 5 cases showed nerve infiltration by chronic inflammatory cells (Figures 1 and 2). 3 cases showed epithelioid cell granuloma (Figure 3) and Langhans giant cell (Figure 4) without caseous necrosis, and Acid Fast Bacilli were kept under tuberculoid form and 2 cases with the absence of granuloma, giant cell, caseous necrosis but positive for AFB (Figure 5) were kept under borderline form of leprosy under Ridley-Jopling scale. 

## 4. Discussion

Leprosy is common disease of India, Nepal, and Myanmar [1]. Although leprosy is a treatable disease, many patients will continue to experience significant nerve damage [1]; Patients with leprosy have high rates (56%) of established nerve damage at diagnosis, which frequently lead to disability [1]. In Nepal, about 7–16% of patients present with this form of leprosy [3].

Diagnosing leprosy relies on the identification of the typical clinical and histopathological involvement of the skin and nerves [1]. The absence of typical dermatological features greatly decreases clinical diagnostic accuracy and necessitates histological confirmation [1]. Skin biopsies from anesthetic areas may fail to show histological changes suggestive of leprosy in cases of pure neuritic leprosy [3]. The diagnosis of primary neuritic leprosy (PNL) and its differentiation from other causes of peripheral neuropathy is difficult, since acid-fast bacilli (AFB) smears and skin biopsy are negative from anesthetic areas [4]. A biopsy of the involved nerve is the only conclusive method of diagnosis [4]. Such a biopsy may not necessarily be free of complications when a large nerve is involved [4]. Nerve biopsy is limited by sampling errors, low sensitivity, and permanent nerve deficit, as still functioning nerves often need to be sacrificed [1]. However, nerve sparing techniques such as FNAC have been shown to maintain a high diagnostic yield when compared with standard biopsy and have less side effects [1]. Since this is a relatively “nerve sparing” procedure, this may allow the examination of motor nerves when sensory nerves are not involved or cannot be sampled [1]. No incidence of iatrogenic loss of motor, sensory function, or of local changes has been reported, following nerve FNAC [2–5]. Extensive medline search did not show any evidence of transmission of leprosy during FNA or nerve biopsy. However, we suggest that precaution should be always taken by using face mask and hand gloves, and one should remain very careful to prevent needle prick in each and every case during the procedure, because the transmission of leprosy by inoculation is well documented in the literature [11]. 

Fine needle aspiration has proved to be a simple technique to demonstrate inflammatory aspirate of lymphocytes, macrophages, epithelioid cells granulomas, Langhans giant cells, caseous necrosis, and AFB from the involved nerves in suspected cases of PNL [2–7]. Schwann cells arranged in a parallel fashion could be seen intimately mixed with granulomas [4]. The procedure is simple and minimally traumatic and has shown to provide valuable information, not only in the demonstration of leprotic inflammation, but also in the categorization of leprous neuritis along the Ridley-Jopling scale [2, 5, 6]. Cases with nerve involvement in leprosy classified leprous neuritis into paucibacillary (PB), borderline borderline (BB), borderline lepromatous (BL), and polar lepromatous leprosy (LL) types [6]. PNL in general will fall from typical tuberculoid to borderline lepromatous leprosy in the Ridley-Jopling classification [3]. A few cases of the Indeterminate [12] and lepromatous [13] form of pure neural leprosy have been also reported. Cytologically, tuberculoid PNL manifests with, either caseous necrotic material or epithelioid granulomas or a combination of both [2, 5]. Cutaneous involvement in leprosy is never associated with caseation, whereas the tuberculoid neuritic form of leprosy presents frequently with caseous necrosis [2, 5]. In the present study, 3 cases fall in tuberculoid and 2 cases in borderline lepromatous form of leprosy and managed accordingly, and caseous necrosis has not been found in any case. Like in its cutaneous counterpart, tuberculoid PNL is characterized by a high degree of cell mediated immunity (CMI) with intense granulomatous neuritis and no AFB [5, 12]. Borderline PNL is associated with a lower degree of CMI with several sites of neurologic impairment, with few, or many, AFB [5, 12]. A lepromatous PNL with low or absent CMI manifests with multiple lesions exhibiting numerous organisms within foamy histiocytes [12]. Indeterminate PNL is characterized by a few hypoesthetic or anesthetic patches, with little or no nerve involvement, few organisms, or no cutaneous changes [12].

 Literature review of cytological findings of skin and nerve aspirates with Ridley-Jopling classification [6, 8–10] is tabulated in Table 2. 

The accuracy of cytological classification along the Ridley-Jopling spectrum in nerve aspirate was found in 92% cases [6]. In the present study, we were able to classify all 5 cases according to criteria devised for interpreting the cytology of nerve aspirates [6]. However, a negative aspirate does not entirely rule out leprosy [6]. A strong concordance in tuberculoid (90%) and in lepromatous (93.7%) cases has been documented [14]. Mid-borderline cases of leprosy show a problem in proper diagnosis [14].

Nerve fragments comprising of Schwann cells cytologically simulate to epithelioid cell granuloma in low-power screening. It can be differentiated by morphological details made in high power. The Schwann cells are spindle-shaped cells of varying sizes with abundant, pale-staining cytoplasm with pulled out ends, and have oval, centrally or eccentrically, placed vesicular nuclei with ill-defined nucleoli [4]. Epithelioid cell granuloma is comprised by the collection of epithelioid cells. The epithelioid cells can be differentiated from Schwann cells by the presence of pale cytoplasm and vesicular elongated, drawn out, indented or folded nucleus, producing a shape reminiscent of a footprint. The nuclear chromatin is fine, and nucleoli are usually inconspicuous. The cytoplasmic margins are indistinct [4]. 

When the nerve involvement is solitary, the differential diagnosis includes tumors of the nerve sheath (neurofibromas and schwannomas), sarcoidosis, and sporotrichosis [15]. In sarcoidosis, the granulomas may be randomly dispersed from the roots to the distal nerve trunks and branches [15]. In these cases, involvement of neural tissue occurs after the expansion of a neighboring granuloma, while in leprosy the granulomas occur primarily in the nerve [15]. Moreover, sarcoidosis usually presents as a multifocal disease with multiple granulomas in several organs, mainly in the lung tissue [15]. The diagnosis of sporotrichosis can be suggested by the occurrence of several abscesses distributed along the lymphatic chains, but with no relation to the neural tissue [15]. In endemic area of leprosy, pure neuritic leprosy should always be considered in the investigation of a peripheral neuropathy [15]. We suggest that FNAC of the nerve will solve the problem to differentiate it from other lesions and can establish the conclusive diagnosis in these cases. FNAC of nerve sheath tumor shows benign spindle cells with palisaded long slender nuclei having pointed ends in fibrillary background. Sarcoidosis shows open granulomas with the absence of necrosis, acute, and chronic inflammatory cells and rarely the presence of asteroid bodies or Schaumann bodies in histiocytes and giant cells. Sporotrichosis shows suppurative granuloma with surrounding plasma cells and demonstration of fungal elements.

## 5. Conclusion

FNAC is safe, early, easy, less invasive, time saving, and cost effective procedure for the diagnosis of pure neuritic leprosy, and biopsy should be reserved only for inconclusive cases.

## Figures and Tables

**Table 1:** Clinicocytological details of the cases of pure neuritic leprosy.

	C/N	Cl/f	Site of FNA	Cytological details								
				cellul	Ner frag	Sch cells	L	M	Epi cell	Gr	Casnecr	AFB
1	26/M	Num,	Rt com per	++	+	+	++	++	+	−	−	++
2	32/M	Sens def	Rt uln	+	+	+	++	++	−	−	−	++
3	45/F	Pain, pares	Lt med	++	++	++	+++	++	++	++	−	−
4	24/M	pain	Rt uln	++	++	++	+++	+	+++	+++	−	−
5	42/M	pain	Lt sup per	++	++	++	+++	+	+++	+++	−	−

C/N, Case Number; M, Male; F, Female; Cl/f, Clinical feature; Num, Numbness; Sens def, Sensory deficiency; pares, paresthesia; fna, fine needle aspiration; Rt, Right; Lt, Left; com per, common peroneal; uln, ulnar; med, median; sup per, superficial peroneal; cellul, cellularity; Ner frag, Nerve fragment; Sch, Schwann; L, Lymphocytes; M, Macrophages; Epi, Epithelioid; Gr, Granuloma; Cas necr, Caseous necrosis; AFB, Acid Fast Bacilli; (Cellularity: +, moderate, ++, good); (Nerve fragments and other cells: +, present; ++, moderate in number; +++; numerous); (AFB: ++, many).

**Table 2:** Cytomorphological classification of leprosy according to Ridley-Jopling spectrum.

Class	Singh et al. [8] (skin smear)	Prasad PV et al. [9] (skin smear)	Jaswal et al. [10] (skin smear)	Vijaikumar et al. [6] (nerve aspirate)
TT	Cellular smears, cohesive epithelioid cell granulomas, numerous lymphocytes not infiltrating the granuloma, no stainable AFB	Cellular material with predominantly lymphocyte population and histiocytes without epithelioid transformation, no stainable AFB	Cellular smears, cohesive epithelioid cell granulomas, numerous lymphocytes not infiltrating the granuloma. BI 0–3+	Good cellular aspirate*·* Cohesive epithelioid cell granuloma or lymphocytic cell collection*·* Predominantly epithelioid cells with predominant to moderate number of lymphocytes. Occasional giant cells and neutrophils*·* BI 0-1+.

BT	Same as TT	Cellular material with lymphocytes, histiocytes and epithelioid cells, foamy macrophages are not a feature, no stainable AFB.	Same as TT	Same as TT

BB			Fair cellular yields, poorly cohesive granuloma composed of an admixture of epithelioid cells and macrophages, few lymphocytes infiltrating the granulomas. BI 1-2+	Fair cellular aspirate*·* Mixed cellularity of predominantly nonfoamy macrophages, moderate number of epithelioid cells and lymphocytes. Macrophage granuloma*·* BI 2-3+.

BL	Moderate cellularity, singly dispersed macrophages with no epithelioid cells. Numerous lymphocytes diffusely scattered along with macrophages. BI 3-4+	Moderate cellularity, singly dispersed macrophages with no epithelioid cells. Numerous lymphocytes diffusely scattered along with macrophages. BI 3-4+	Moderate cellularity, singly dispersed macrophages with negative images, no epithelioid cells, numerous lymphocytes diffusely admixed with macrophages. BI 3-4+	Fair cellular aspirate*·* Predominantly lymphocytes and moderate number of foamy macrophages. BI. 4-5+.

LL	Heavy cellularity, numerous foamy macrophages in fatty background with a few lymphocytes. BI 5-6+	Heavy cellularity, numerous foamy macrophages in fatty background with a few lymphocytes. BI 5-6+	Heavy cellularity, numerous foamy macrophages in fatty background with intracellular and extracellular negative images, few lymphocytes. BI 4–6+	Fair to poor cellular aspirate*·* Predominantly foamy macrophages and few lymphocytes*·* BI 6+

TT, tuberculoid; BT, borderline tuberculoid; BB, borderline borderline; BL, borderline lepromatous; LL, lepromatous leprosy; BI, Bacillary index.
